# Influence of COVID-19 on Tertiary Orthopaedic Centres

**DOI:** 10.7759/cureus.31388

**Published:** 2022-11-11

**Authors:** Kashyap Kanani, Ratnakar Ambade, Aditya Pundkar, Rameez R Bukhari, Rohan Chandanwale

**Affiliations:** 1 Orthopaedics, Jawaharlal Nehru Medical College, Datta Meghe Institute of Medical Sciences, Wardha, IND

**Keywords:** covid-19 effect on health services, health care professionals, trauma in covid-19, pandemic effect, indoor orthopaedic practice, covid-19 pandemic

## Abstract

Coronavirus disease 2019 (COVID-19) is a highly contagious lethal infection that has successfully spread all across the world. The novel coronavirus that is behind the menace and spread of COVID-19, is the next in the lineage of the *Coronaviridae* family of viruses, which had previously given two deadly viruses with limited geographical extent. After sustaining for more than two years, the virus is still active and keeps on mutating to evade human immunity. The impact of COVID-19 is felt not only by patients of COVID-19 who go through the trauma but also by non-COVID-19 patients due to the non-pharmacological interventions (NPIs) enforced. Patients in the orthopedic departments suffered a huge blow as their rehabilitation practices were stalled due to a lack of health professionals and also restrictions imposed. But to soften the blow, usage of telemedicine was done in some instances so that the essential therapies can continue despite the movement restrictions imposed. COVID-19 has disrupted many aspects of human life including clinical practices and this endeavor is to review those aspects and provide conclusions if any. The aim of the study is to review the available resources regarding Indoor orthopedic practice during the COVID-19 pandemic and draw a conclusion that can help further research on the aforementioned topic.

## Introduction and background

Coronavirus disease 2019 (COVID-19) evolved into a deadly pandemic that grappled the whole world for more than two years. Since the start of the virus with patient zero registering from the Chinese city of Wuhan, Hubei province, the virus has spread to more than 200 territories and regions of the world and registered infection instances stood at 529,555,478 while the loss of lives owing to its complications stood at 6,289,588 as of June 1, 2022. Even after a considerable amount of time, it is very much prevalent and various healthcare agencies are issuing time-to-time advisories about the newer variants and mutations in the novel coronavirus that causes the deadly disease [1-3]. The previous members of the *Coronaviridae* family of viruses were not as deadly as the current one, which has spike proteins that get attached to the angiotensin-converting enzyme 2 (ACE 2) receptors, which facilitate the entry of the virus into the human cell. The incubation period of the viral disease is about two to 12 days averaging at six days.

Symptoms include wide-ranging aspects from cough, cold, and fever to dyspnea and the need for sophisticated medical intervention due to worsening of the condition. The fact that more than five million people died due to the complications created by COVID-19 is frightening. COVID-19, in recent years, is the sole event in human civilizational history that has had wide spectral impacts on human lives. From anatomical to socioeconomic, all the aspects of life were severely affected across the world. The geographical extent of the previous two similar epidemics was somewhat restricted to certain areas but COVID-19 transgressed all the existing boundaries. The impact was felt not only by the persons infected by COVID-19 but also by individuals not infected by COVID-19 [4]. Orthopaedic patients comprised one of the worst affected categories of non-COVID-19 patients affected by the pandemic. Rehabilitative care is one of the most important factors in the treatment and healing procedures of orthopaedic patients. But COVID-19 induced movement restrictions and lockdowns along with the diversion of healthcare resources like healthcare professionals solely for containing COVID-19, which made the life of an orthopaedic patient more miserable [5].

Several measures were taken to contain the pandemic. These measures proved a mixed bag of results. On environmental fronts, due to almost no vehicles on the road except the emergency ones, pollution levels were drastically lowered and people experienced a much cleaner environment [6,7]. Animals were reclaiming their lost share of biodiversity by coming into the city limits. But not all impact was good. During the pandemic, almost all resources were dedicated to the containment of the spread of the deadly virus and its treatment. This created a scarcity of resources for non-COVID-19 patients as they were left hanging in the middle. For various chronic diseases like hypertension, diabetes, renal failure, cardiovascular disease (CVD), and orthopaedics, patients need regular follow-up visits with doctors or clinicians. Due to the imposition of movement restrictions, difficulties for all these patients increased manifold [8,9]. The study gauges the impact on orthopaedic practices, which is indoor in nature, during the COVID-19 pandemic. Various studies were reviewed and the gist is presented here along with conclusions.

Search strategy

The present comprehensive review was conducted using Preferred Reporting Items for Systematic Reviews and Meta-Analyses (PRISMA) guidelines, addressing the effect of COVID-19 on indoor orthopaedics practice. Data resources of PubMed/MEDLINE, Embase, Science Direct, Cochrane Library, and site of clinical trials were consulted for studies published until May 2022. The results were filtered for studies written in the English language. The words that were used for the search strategy on various databases were COVID-19 and orthopedics and non-COVID-19 patients. The literature search on PubMed/MEDLINE was based on the terms “effect of COVID-19 on indoor orthopedics practice” [All Fields] AND “non-COVID-19” [MeSH Terms] OR “indoor orthopedic practices” [All Fields]. The abovementioned words (effect of COVID-19 on indoor orthopaedics practice and indoor orthopedic practices) were used in the search strategy on the Cochrane Library, the database for systematic review.

Selection criteria

**Figure 1:**
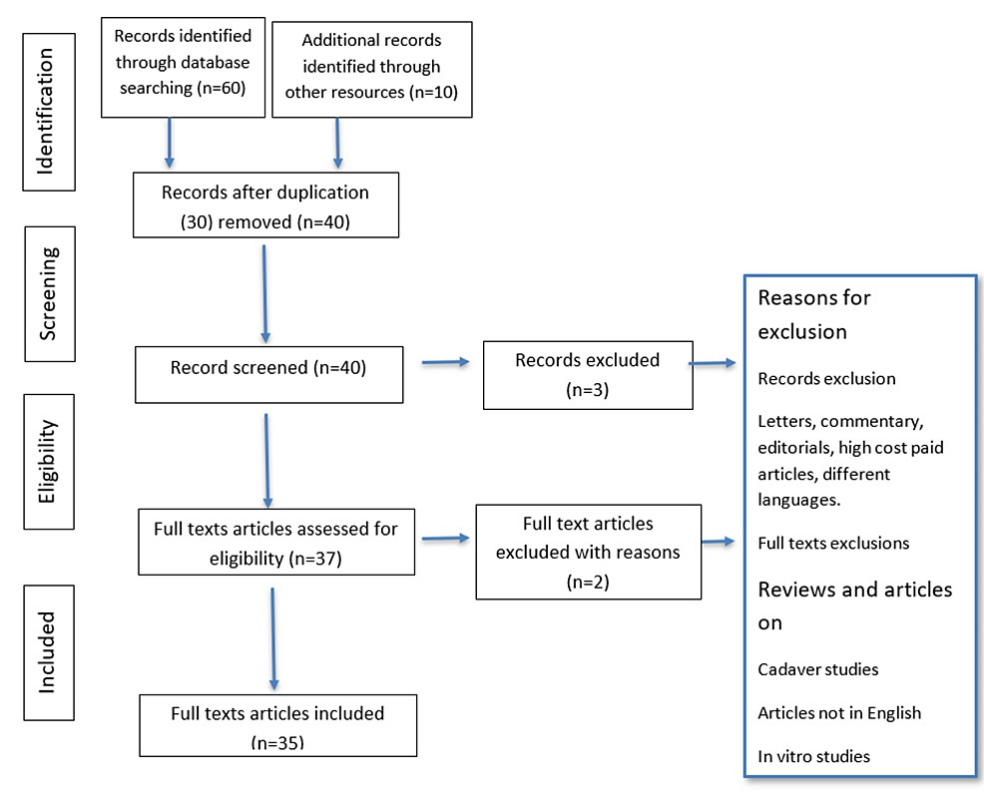
Study selection process using the Preferred Reporting Items for Systematic Reviews and Meta-Analysis (PRISMA) guidelines

The selection criteria included systematic reviews, randomized and non-randomized controlled trials in humans, and retrospective and prospective cohort studies. Studies on the correlation between the COVID-19 situation and its impact on orthopaedic patient practice were included. Technical reports, animal studies, cadaver studies, in vitro studies, case reports, letters to the editor, and review papers were not included (Figure 1).

Study selection

Three of the reviewers screened all identified titles and abstracts independently. In addition, the reference lists of the selected abstracts and the references of systematic reviews, randomized and non-randomized controlled trials (human), and retrospective and prospective cohort studies were manually searched. The full text was accessed for studies appearing to meet the selection criteria or for which unsuitable data in the abstract and title was available.

## Review

Orthopaedics and indoor practices

Orthopaedics mainly deals with muscle skeleton formation also known as the musculoskeletal system. It consists of muscles, bones, tendons, ligaments, and various joints. Various problems related to the above-mentioned parts of the human body arise during the course of a lifetime. Age-related issues come with weakness in joints and bones, athletes often come across certain injuries and deformation, long-term joint pain, and so on. All these deformities and injuries are dealt with under the branch of medicine known as orthopaedics. These injuries and pains are quite prevalent and the majority of the population has to visit orthopaedics clinics for one reason or the other [10,11]. These injuries or pain are treated either through surgical means if required or through non-surgical means. Generally, the orthopaedist deals with a wide variety of joint pains, bone fractures, back and neck pain, and certain congenital issues. After a diagnostics test, which can include one or more of many tests such as CT scan, MRI scan, bone scan USG, etc. After pinpointing the problem, the next step is to medically intervene either surgically or non-surgically. X-ray is the most common methodological test through which clinicians diagnose any fracture or dislocations and act accordingly. If the injury is small and mere inflammation can be observed and diagnosed then the patients are suggested to take anti-inflammatory medications, which lower the inflammation that could be possibly due to the tearing of tendons or muscle [12,13].

In the case of surgical interventions, the patient needs to be hospitalized and there is a need for sophisticated medical care. In case of post-surgery mild injuries or pain, non-surgical interventions such as indoor practices which includes wide range of movements and manoeuvre, only highly skilled health professionals are required. The setting of practice can either be a clinic or the patient's home. But as COVID-19 mandated the closure of almost all the facilities other than COVID-19 care facilities, orthopaedics patients found it difficult to continue or seek the necessary help they require. Many non-essential surgeries were postponed as healthcare professionals were already overburdened with the huge influx of COVID-19 patients [14-16]. The statistics from the outpatient departments (OPDs) of various hospitals proved that the admissions in the department came down to almost nil as fear was instilled in the patients' minds that they would contract the infection if they went out. The section-wise admissions were also telling the same stories. As the whole world came to halt, all the sports-related events were called off or postponed and sportspersons were not allowed to practice outdoors as the venues where these sportspersons practised were closed. Hence, almost no sports injury cases were reported. Referrals obtained from external sources also saw a marked decrease after the start of the COVID-19 pandemic. Most patients requiring the medical intervention of level one trauma centres were transferred to level two trauma centres. Level one trauma centres generally deal with polytrauma and open injuries. However, in case of severe injuries requiring multispectral surgeries, level one trauma units were called for interventions. In one study done in a setup situated in England, the demographic pattern along with the injury pattern was studied. Although the study was in a limited setup, it shows an almost constant split in the influx of male and female patients pre- and post-pandemic. According to the study, the percentage of people who reported lower limb injuries prior to the arrival of the pandemic was 33% while upper limb injuries were 30%. Together they accounted for more than two-thirds of all the patients reported while in post-pandemic setup, the injuries remained the same but the occurrence fell to half from 66% to 33% [17-19]. Fractures in the hip regions generally occur indoors or in enclosed spaces and the fall is not as severe. The fall associated with the hip fracture is often a low-injury fall. Hence, the movement restrictions and lockdowns had little to no impact on the cases registered under the hip fractures category. Diametrically opposite is the scenario related to polytrauma where the fall is a high-intensity fall and generally considered as a fall greater than 1.5 meters. In fact, the number of registered cases under this category decreased. The reasons behind such an outcome are attributed to the exemption to the construction activities. Also, lockdowns and movement restrictions mandated people to stay at home. Helps and maids were not allowed and hence many tasks were done by the family members resulting in injuries. The fall suffered due to geriatric care were independent of the non-pharmacological interventions (NPI’s) like lock down and physical distancing [20-22].

Among the orthopaedic professionals, certain dos and don’ts were circulated as norms to safely work in the COVID-19 scenario. Every patient had to be treated as COVID-19 positive even if it is not being claimed or asymptomatic. All patients admitted for any reason needed to undergo screening tests for COVID-19. All preventive measures and safeguards needed to be followed in order to protect healthcare professionals from getting infected. However, the cases registered under orthopaedics OPD were of casualty only as people were afraid of contracting COVID-19. Minor injuries were being managed over a call and ad hoc measures on the line of do-it-yourself were being employed. The major contributor to orthopaedics-related injuries is road traffic mishaps. During the pandemic, with lockdown and movement restrictions measures enforced, almost no vehicles other than emergency vehicles were permitted on road. Hence, there was a sharp fall in accident-related orthopaedic OPD entries [23-25]. 

In the case of patients recovering or rehabilitating from the previous grave injuries of bones, muscles, tendons or ligaments, they were mandated to stay at home as this was categorized as a non-essential medical scenario. Orthopaedics departments all over the world were busy devising the optimized protocol for the treatment of orthopaedics patients during the COVID-19 pandemic. Health professionals were playing both roles, first as the front-line warriors in the fight against COVID-19 and second in their respective fields of specialization as certain emergencies among non-COVID-19 patients mandated them to do so [26-28].

The importance of physical exercises and various manoeuvring practices is immense in the case of patients with sprained joints, traumatic injuries pertaining to muscle, bones, tendons as well as ligaments, fractures in bones, and upper as well as lower limb injuries. The rehabilitation phase is as important as the operative or treatment phase as old injuries can aggravate or renew after a certain time interval if not taken care of properly. Such rehabilitation practices and exercises must go on until the maximum possible functions of the affected part are regained. Cervical and spondylitis of the lumbar region is a widely prevalent chronic condition, which is symptomatic in half of the cases and is quite difficult to handle. Hence, proper attention is needed to recover or improve the existing condition. During the COVID-19 pandemic, home visits or clinic visits were not happening as earlier but the process of rehabilitation was essential. Many patients discontinued the process and this will cause a greater challenge in near future. But several orthopaedics patients who needed less specialised help adapted to the situation and started consulting on the telephone or online. Prior to the non-pharmacological interventions like lockdown and movement restrictions, orthopaedic patients were able to seek health care services. After the imposition of such restrictions, seeking healthcare services other than for COVID-19 was difficult. This has created difficulties among orthopaedic patients as well in seeking healthcare services [29-31].

Rehabilitation exercises must be regular and consistency must be maintained as it prevents the stiffness in muscles, which in long turn hurts the well-being of the patient and also affects the quality of life. Osteoporosis, which was earlier considered an ailment affecting mostly the geriatric age groups is now being seen among young adults and the middle-aged population too. The main causes behind it are lack of proper physical exercise, indoor work culture, sedentary lifestyle, and not taking adequate amount of sunlight, which stimulates the absorption of vitamin D that strengthens the bones. In times of COVID-19, where restrictions mandated staying at home, people avoided check-ups for minor issues and early diagnosis of osteoporosis was not possible, which could further increase its effect. As time increases, the chances of bone injury become high and full recovery is possible in most cases [32,33].

The essentiality of indoor orthopaedic practices was understood by the patients and they consulted with healthcare professionals for regular medical intervention via the internet and video conferences. Many of the exercises are repetitive and simple and do not need to be performed by doctors. Such exercises were instructed via internet telephony and patients followed routines with the assistance of their family members and near ones. This somewhat helped in tough times as there were no other feasible alternatives available [34-36]. However, elemedicine was largely limited to the tech-savvy and younger section of the patients. Also, technical know-how about telemedicine technology was the prime barrier to accessing health care services via the internet [37].

## Conclusions

Although new orthopaedic patient entries were reduced, the rehabilitative treatment was hampered by COVID-19. Serious and grave injuries were treated at the hospital facility with limited resources after following tedious protocols to ensure that the infection of COVID-19 does not spread to other patients as well as treating doctors. Non-serious rehabilitative patients were completely left in the lurch. Telemedicine was one such option available to orthopaedic patients but it was largely limited to the tech-savvy and younger section of the patients. Also, technical know-how about telemedicine technology was the prime barrier to accessing health care services via the internet.
